# Integration of an anellovirus genome in the SKNO-1 acute myeloid leukemia cell line

**DOI:** 10.1128/spectrum.00904-26

**Published:** 2026-08-31

**Authors:** Na Cui, Stephanie Goya, Eli A. Piliper, Alexander L. Greninger

**Affiliations:** 1 Department of Laboratory Medicine and Pathology, University of Washington School of Medicine, Seattle, Washington, USA; 2 Vaccine and Infectious Disease Division, Fred Hutchinson Cancer Research Center, Seattle, Washington, USA; University of Manitoba, Winnipeg, Manitoba, Canada

**Keywords:** anellovirus, SKNO-1, acute myeloid leukemia, myeloblast, torque teno virus, culture, integration

## Abstract

Anelloviruses are highly diverse, ubiquitous single-stranded DNA viruses whose replication, cellular reservoirs, and mechanisms of persistence remain poorly understood. Here, we identify and characterize an *Alphatorquevirus homin29* genome stably integrated into an rDNA locus of human chromosome 21 in the acute myeloid leukemia cell line SKNO-1. Large-scale mining of NCBI Sequence Read Archive data sets revealed unusually high anellovirus k-mer abundance specifically in sequencing runs of the SKNO-1 cell line from multiple laboratories. *De novo* assembly of RNA-Seq data recovered a 3.25 kb viral genome, and long-read PacBio sequencing confirmed its integration within the *RNA45SN2* gene on human chromosome 21. Digital droplet PCR (ddPCR) quantified ~0.5 viral genomes per cell, and RT-ddPCR detected transcripts from ORF1 and viral integration-associated flanking repeats, consistent with transcription of the integrant. The ORF1 coding sequence encoded a truncated but structurally conserved capsid protein retaining jelly roll and P-domain features, but lacking the C-terminal domain. Analysis of public ChIP-Seq data from the SKNO-1 cell line demonstrated broad occupancy of the BRD4 transcriptional coactivator across the viral genome, along with focal enrichment of hematopoietic ETS family transcription factors within the integrant’s ~300 bp untranslated region upstream of viral open reading frames. Together, these findings demonstrate stable integration, chromatin accommodation, and transcriptional maintenance of an *Alphatorquevirus* in a human leukemia cell line, providing a unique model to study host tolerance and transcriptional regulation of anellovirus DNA, and informing our understanding of anellovirus hematopoietic cell tropism.

**IMPORTANCE:**

Anelloviruses are a curious group of single-stranded DNA viruses with substantial genetic diversity that have been found ubiquitously among humans and are hypothesized to be a potential commensal human virus. Their omnipresence has been matched only by the dearth of our understanding of their basic biological processes—how they persist, how they replicate, and how they spread. Here, we identify a naturally integrated *Alphatorquevirus* genome in the widely used AML cell line SKNO-1 and show that it is stably maintained, transcribed, and embedded within a rDNA locus on human chromosome 21. This discovery highlights the ability of anelloviruses to integrate into human DNA, generates hypotheses around transcription factors used in anellovirus gene transcription, and suggests anelloviruses can persist in myeloblasts.

## INTRODUCTION

Anelloviruses comprise a highly diverse group of small, circular, single-stranded DNA viruses that are ubiquitous in humans yet poorly understood in terms of their biology and clinical significance. Since their discovery in 1997, anelloviruses have been detected in approximately 90% of adults and are most commonly found in blood and bone marrow (1). Viral DNA has also been identified in multiple human tissues and body fluids, including ovary, lung, saliva, and respiratory secretions (2, 3).

Advances in viral metagenomics have expanded the known diversity of human anelloviruses and renewed interest in their diagnostic and therapeutic potential (3). Studies of transfusion- and transplantation-associated infections have demonstrated that donor-derived anellovirus DNA can persist in recipients for several months (4, 5). Moreover, circulating anelloviruses have been proposed as potential biomarkers for organ transplant rejection (6–9). Despite these associations, the mechanisms underlying anellovirus persistence, replication, and immune modulation remain largely unclear (10–12).

Anelloviruses exhibit substantial genetic diversity approaching that of HIV-1 (4). As of 2025, the International Committee on Taxonomy of Viruses (ICTV) classifies the *Anelloviridae* family into 34 genera and 173 species identified across diverse mammalian hosts (13–17). Human anelloviruses have traditionally been assigned to three genera: *Alphatorquevirus* (Torque teno viruses, TTV), *Betatorquevirus* (Torque teno mini viruses, TTMV), and *Gammatorquevirus* (Torque teno midi viruses, TTMDV) (3), although recent metagenomic sequencing data indicate that additional anellovirus genera are present in humans (18, 19). Members of the *Anelloviridae* family are non-enveloped and contain a 2.0–3.9 kb negative-sense circular DNA genome encoding six open reading frames (ORFs) through alternative splicing (20–22). Because they lack a viral DNA polymerase, anelloviruses depend on host replication machinery, possibly replicating in the cell nucleus through a rolling-circle mechanism, although their mechanism of replication remains unclear (10, 23). Although human anelloviruses have been detected in a variety of tissues, the primary cellular reservoir supporting active replication remains uncertain. Evidence from previous studies suggests replication may occur in bone marrow cells and activated peripheral blood mononuclear cells (24, 25). However, progress in defining viral tropism and replication dynamics has been constrained by the absence of efficient cell culture systems or suitable animal models. To date, most insights have been derived from plasmid-based transfection studies in human cell lines such as COS1, MOLT4, HEK293, 293TT, and L428, which do not support productive viral replication (21, 26).

In this study, we describe the discovery of an *Alphatorquevirus* in the human acute myeloid leukemia (AML) cell line SKNO-1. Remarkably, the viral genome was integrated into the host *RNA45SN2* gene on chromosome 21 and stably transcribed and maintained in the cell line. Through combined bioinformatic and experimental approaches, we confirm the presence and transcriptional activity of this anellovirus and use existing systems biology data sets generated from this cell line to inform host cell tropism and anellovirus transcription.

## RESULTS

### Anellovirus k-mers are primarily detected in public sequencing data from SKNO-1 cells

**Fig 1:**
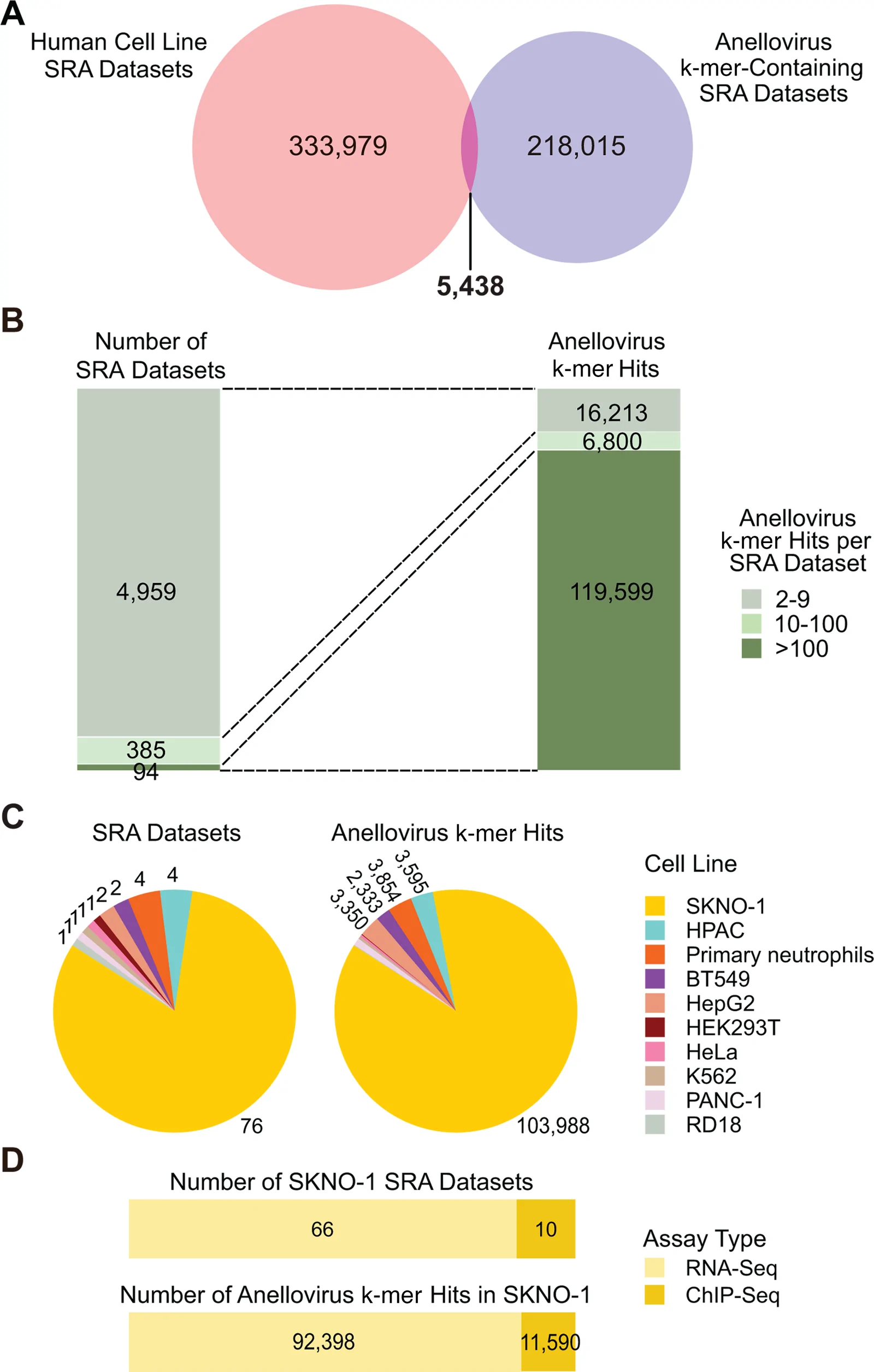
Data mining of NCBI SRA data sets for human cell lines with anellovirus k-mer hits. (**A**) Venn diagram illustrating the overlap between human cell line SRA data sets and those containing anellovirus k-mers. (**B**) Correlation analysis of SRA data sets with anellovirus k-mer hits, categorized by hit frequency: low (2–9 k-mers), medium (10–100 k-mers), and high (>100 k-mers). The dotted lines connect each hit-frequency category to its corresponding total number of hits. (**C**) Classification of anellovirus k-mer-enriched SRA data sets by human cell line type. (**D**) Overview of sequencing methodology for anellovirus k-mer-rich SKNO-1 SRA data sets.

We investigated the presence of anellovirus in public sequencing data by querying the Cloud-based Taxonomy Analysis Table on NCBI SRA using BigQuery. This search yielded 218,015 SRA data sets with at least two anellovirus k-mer hits of 32 nt in length (Table S1). To identify a potential model cell line for anellovirus studies, we cross-referenced the SRA accessions with a collection of the 333,979 SRA data sets annotated as a human cell line (HCL) (Table S2). We identified 5,438 overlapping HCL data sets that contained at least two anellovirus k-mer hits (Table S3; Fig. 1A). Of these HCL data sets, the vast majority (4,959/5,438) had only 2–9 anellovirus k-mer hits, while 385 contained 10–100 hits, and notably, 94 had >100 k-mer hits (representing just 1.7% of the data sets), contributing 83.9% of the total anellovirus k-mer hits (119,599/142,612; Fig. 1B). Strikingly, 76 of the 94 anellovirus k-mer-rich HCL data sets were derived from the SKNO-1 cell line, an FAB-M2 *t* (8, 21) AML cell line established in 1990 from the bone marrow of a 22-year-old man (27), accounting for 86.9% (103,988/119,599) of all anellovirus k-mer hits in these samples. The remaining anellovirus-rich HCL data sets came from other human cell lines, including human pancreatic cancer PANC-1/HPAC (five data sets, 4,780 hits), primary neutrophils (four data sets, 3,854 hits), HepG2 (two data sets, 3,350 hits), BT549 (two data sets, 2,333 hits), K562 (one data set, 394 hits), HeLa (one data set, 390 hits), unknown (one data set, 264 hits), HEK293T (one data set, 141 hits), and RD/18 rhabdomyosarcoma (one data set, 105 hits) (Fig. 1C). Of the 76 SKNO-1 data sets with high anellovirus k-mer counts, 66 were derived from RNA-Seq libraries, representing 88.9% of the total SKNO-1 anellovirus k-mer hits (92,398/103,988), while the remaining 10 were ChIP-Seq (chromatin immunoprecipitation followed by sequencing) libraries, contributing 11.1% of SKNO-1 anellovirus k-mer hits (11,590/103,988; Fig. 1D).

To assess the potential role of contamination in these findings, we examined the origins of these anellovirus-rich data sets. The majority of RNA-Seq runs (41 FASTQ files) were derived from the University of Miami, which studied the enzyme co-activator CARM1 in normal and malignant hematopoiesis (28) and the transcriptional modulator DPF2 in inflammation (29). Other sequencing data were generated by the University Medical Center Mainz (24 RNA-Seq, four ChIP-Seq runs), where SKNO-1 was used in studies on oncogene regulation during acute resistance to BET inhibitors (30). In addition, six ChIP-Seq runs originated from the Broad Institute, focusing on transcriptional regulation associated with KMT2A-rearranged acute myelogenous leukemia (31), and one RNA-Seq run originated from the University of Virginia, comparing transcriptomic profiling of AML primary samples and cell lines (32).

Searching SRA Cloud for sequencing runs with more than one anellovirus k-mer that were also annotated with “SKNO” yielded 138 additional sequencing runs, including 93 RNA-Seq, 41 ChIP-Seq, three ATAC-Seq (assay for transposase-accessible chromatin using sequencing), and one NA-Seq (nuclease accessibility sequencing) (Table S4). Our initial SRA screen suggested both anellovirus DNA and RNA were present in the SKNO-1 cell line across a variety of laboratories from around the world.

### Assembly of anellovirus sequence from SKNO-1 cell line short-read sequencing

To create initial assemblies of anellovirus sequences from the SKNO-1 cell line, we used four different RNA-Seq runs of SKNO-1 cells with the highest number of anellovirus k-mers (Table 1), given that there were no classical DNA-Seq runs available for SKNO-1. Specifically, we used a strand-specific RNA-Seq run of SKNO-1 cells transduced with shRNA-Scramble from the University of Miami in 2018 (5,310 k-mers, SRA accession SRR6008465); an RNA-Seq run of BETiIC90-resistant SKNO-1 cells after 24 h of DMSO treatment from the University Medical Center Mainz in 2022 (1,961 k-mers, SRR22306486); and an RNA-Seq run of SKNO-1 cells from the University of Virginia in 2024 (1,392 k-mers, SRR24085425) (28, 30, 32). Based on a literature review, we also included an RNA-Seq run of SKNO-1 cells published by the German cell line repository at Leibniz-Institute DSMZ (ERR3003594) in 2019 that had 559 anellovirus k-mers but was not annotated as “cell line” and therefore was not included in our initial screen described above (33).

**TABLE 1:** Assembly of anellovirus genome from four SKNO-1 RNA-Seq SRA data sets^*a*^

SRA accession number	SRR6008465	SRR22306486	SRR24085425	ERR3003594
Instrument	NextSeq 500	NovaSeq 6000	NovaSeq 6000	HiSeq 2500
Submission Year	2018	2022	2024	2019
Submitter	University of Miami, USA	University Medical Center Mainz, Germany	University of Virginia, USA	Leibniz-Institute DSMZ, Germany
Reference	(28)	(30)	(32)	(33)
Sequenced fragments/spots	54,934,628	44,374,157	20,228,673	34,619,305
Read format	2 × 75 bp	2 × 150 bp	2 × 151 bp	2 × 151 bp
STAT anellovirus k-mers	5,310	1,961	1,392	559
CZID anellovirus reads (rpm) file count	14,295 (130.1)	3,448 (38.8)	4,095 (101.2)	1,490 (21.5)
Longest *de novo* contig length	3,322 bp	2,976 bp	3,375 bp	3,331 bp

^*a*^ rpm, reads per million.

To ensure specificity, we used the CZID metagenomic pipeline to identify anellovirus-aligning reads (34). Across the four samples, 93.5–97.3% of reads were classified as human, with the only non-human viral genus being *Anellovirus*, except for *Lentivirus* in SRR6008465, which was used as a vector for shRNA-Scramble transduction. We performed *de novo* assembly of the anellovirus-specific reads, yielding one to three contigs per data set. The longest contigs ranged in length from 2,976 to 3,375 bp, aligning with 100% nucleotide identity over a 2,897 bp core region. Upon inspecting the alignment of the longest contigs, we noted that the RNA-Seq assembly from the DSMZ data set yielded a perfect 77 bp overlap between the ends of the longest contig. We trimmed the 5′-end 77 nt overlap and used the resulting 3,254 bp assembly as a reference genome to map reads from the other RNA-Seq data sets analyzed. Mapping the reads from four separate RNA-Seq data sets showed that 99.7–100% of the reads classified as anellovirus perfectly aligned to the reference genome, with no significant nucleotide variability. This finding strongly suggests that there was no viral diversity in the analyzed samples, and a single anellovirus strain was present.

Interestingly, at the 5′ end of the alignment, we observed two distinct populations of reads: one aligned fully to the anellovirus genome, while the other was chimeric, showing a mix of human 28S rDNA gene (human chromosome 21, CT476837.18) at the 5′ end and anellovirus sequence at the 3′ end (Fig. S1). At the 3′ end of the genome alignment, all anellovirus reads showed identity with the reference sequence but included soft-clipped sequences ranging from 30 to 92 nt that were identical to the 5′ end of the reference genome. In addition, we identified two single-nucleotide variants (SNVs) in the assembly at positions 150 (c150a) and 162 (g162a), which were associated with reads that also displayed a 3′-end soft-clipped sequence extending up to 90 nt, beginning at nucleotide 172 of the anellovirus genome (Fig. 1). These soft-clipped sequences showed 100% identity with an LTR-retrotransposon sequence in human chromosome 4 (AC107058.4), suggesting the presence of more chimeric sequences with human genomic material.

These findings were consistent across all four RNA-Seq runs, supporting the hypothesis that an integration event could be responsible for the observed chimerism. We also noted a coverage drop beginning at nucleotide 172 of the viral genome, which was associated with the chimeric reads aligning to human genomic sequences in this region. Finally, we examined RNA-Seq data from other non-SKNO-1 cell lines with anellovirus reads (Fig. 1C), aligning them against both the human genome and their longest anellovirus *de novo* contig and searching for virus-human chimeric reads. While we confirmed the presence of different anelloviruses in these data sets, we did not observe the same pattern of virus-human chimeric reads, suggesting that this feature is specific to the anellovirus in the SKNO-1 cell line.

### Long-read sequencing of the SKNO-1 anellovirus integrated into human chromosome 21

**Fig 2:**
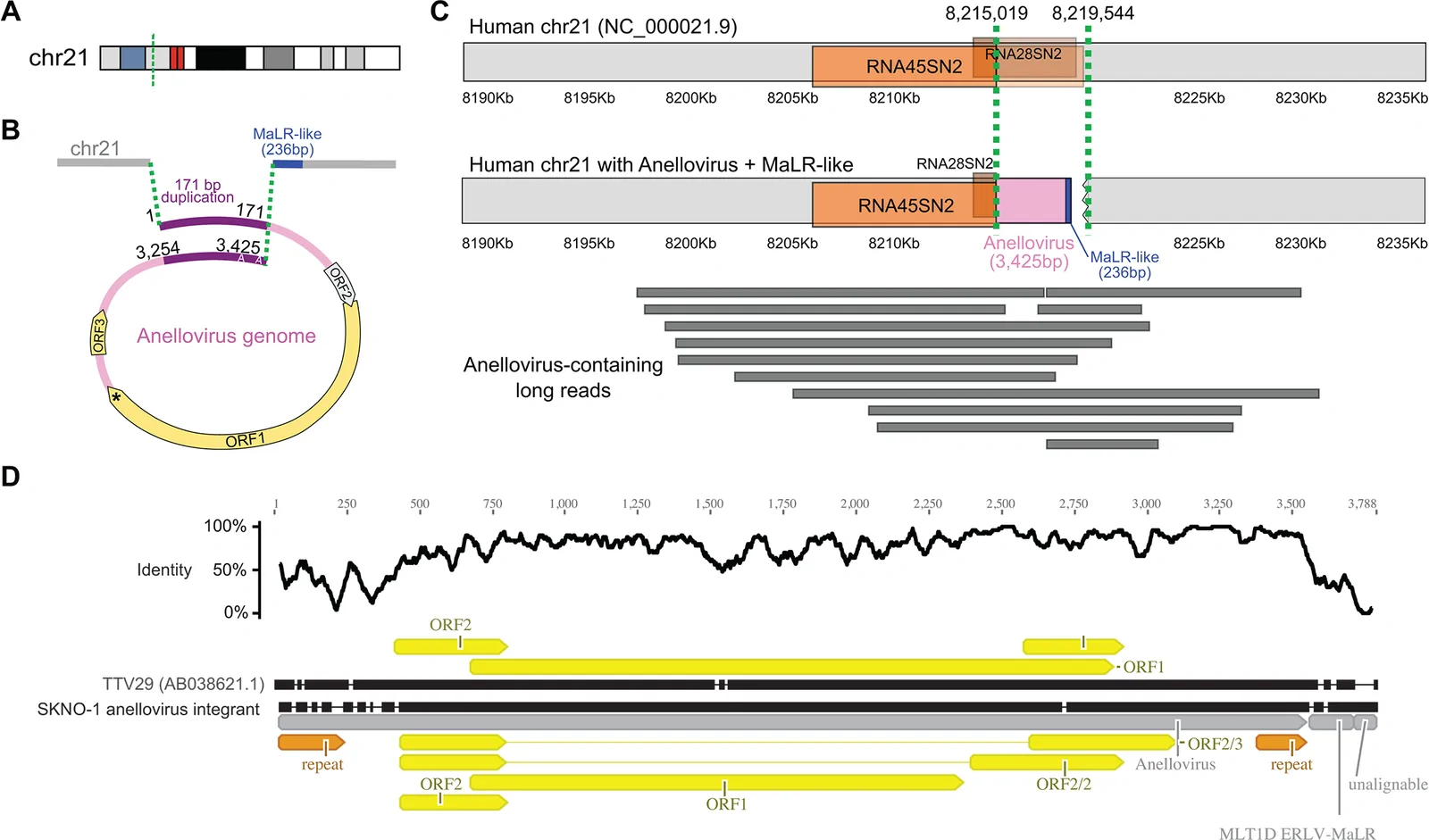
Integration of the anellovirus genome within chromosome 21 of the SKNO-1 cell line. (**A**) Schematic representation of chromosome 21 in the SKNO-1 cell line, showing the integration site of the anellovirus genome (dotted green line). Red represents the centromere, blue indicates the repetitive region, and the grayscale shading indicates Giemsa bands (darker shades correspond to higher heterochromatin and greater AT-rich regions). (**B**) Detailed schematic of anellovirus integration within chromosome 21. The pink region denotes the anellovirus genome, with arrows indicating open reading frames (ORFs). A gray arrow highlights ORF2 containing an early stop codon at position 2, while an asterisk (*) marks the truncation of ORF1, preventing overlap with ORF3. The violet line highlights the duplicated sequences located at both termini of the viral genome, which differ by two point mutations (C→A and G→A), while the blue region indicates an additional nucleotide segment corresponding to a MLT1D ERVL-MaLR-like element. (**C**) Comparison between wild-type chromosome 21 and the chromosome 21 with the anellovirus genome integration. The integrant interrupts the *RNA45SN2/RNA28SN2* rRNA gene. Dotted green lines indicate the region that has been altered within chromosome 21, including specific coordinates for the integration site. Below the schematic of chromosome 21 with the integration, PacBio long reads are shown (represented by gray rectangles), confirming the presence of the integrant in the SKNO-1 J subline. (D) Pairwise alignment of the full SKNO-1 integrant including MLT1D ERVL-MaLR-like sequence, with Torque teno virus 29 with a 50 bp sliding-window nucleotide identity graph. Viral coding sequences using the ACG start codon for ORF1, along with 171 bp viral integration-associated flanking repeats, are depicted.

To confirm the potential integration of an anellovirus in the SKNO-1 cell line, we obtained SKNO-1 cells from the DSMZ repository in Germany (G subline) and the JCRB repository in Japan (J subline) and performed 40× PacBio sequencing of high-molecular-weight dsDNA isolated from these cell sublines. We obtained 8,683,919 reads with an N50 length of 16,563 bp from the G subline and 8,366,596 reads with an N50 length of 18,656 bp from the J subline. Mapping these PacBio reads to the draft assembled anellovirus genome from DSMZ RNA-Seq revealed five reads (median length, 17,024 bp) for the G subline and 12 reads (median length, 18,957 bp) for the J subline. Aligning the anellovirus-mapping PacBio reads to each other recovered a 3,425 bp anellovirus sequence that consisted of the 3,254 bp draft DSMZ anellovirus genome assembled from RNA-Seq reads, followed by a repeat of the 5′ end 171 bp of the contig at the 3′ end (Fig. 2A and B). These repeats contained the two single-nucleotide variants—c150a and g162a—which were previously identified in our RNA-Seq assembly.

Further analysis revealed that the anellovirus genome was flanked by human sequences. NCBI BLAST searches identified these sequences as being part of human chromosome 21, specifically within the rDNA genes *RNA45SN2* and *RNA28SN2* (Fig. 2C). The full integration region spanned from 8,215,019 to 8,219,544 on human chromosome 21 (NC_000021.9). The integrated sequence consisted of the 3,425 bp anellovirus genome, 10 bp at the 3′ anellovirus-human junction that could not be further characterized, 146 bp that perfectly matched the negative strand of human chromosome 4 (coordinates 65,017,569–65,017,714, NC_000004.12, matching a Medium Reiterated Frequency Repeat 1 subtype D [MLT1D] repeat in the Endogenous Retrovirus-Like/Mammalian Apparent LTR Retrotransposons [ERVL-MaLR] family), and 80 bp that could not be aligned to anything in the nt/nr database by BLASTn. Overall, the integration replaced 4,526 bp of human sequence with a 3,661 bp integrant.

The structure of the integrated viral genome was supported by contiguous read alignments extending up to 17,719 bases at the 5′ end and 11,263 bases at the 3′ end of the viral sequence (Fig. 2C). Notably, the integration junctions were identical across both G and J sublines. The 5′ junction occurred at a homopolymeric stretch of eight guanosines within chromosome 21, resulting in the loss of one human G-quadruplex motif. This event left a single G-quadruplex element at the beginning of the integrated genome, followed by the 171 bp viral integration-associated flanking repeat at the 3′ end. In addition, two of the 12 anellovirus-containing reads from the J subline extended over 12 kb upstream of the integration site on human chromosome 21 and captured only the first 308 nucleotides of the anellovirus integrant and then continued for 2,592 nt on chromosome 21, thus omitting the rest of the anellovirus genome and the MLT1D ERVL-MaLR sequence.

To further explore the integration, we analyzed the depth of sequencing coverage for the anellovirus and several housekeeping genes (Fig. S2). In both G and J sublines, we observed overall consistent chromosomal coverage with slight variations that reflect the underlying karyotypic heterogeneity of the SKNO-1 cell line (Table S5). Specifically, *SDHA* (chr5) showed elevated depth (~68×), consistent with the known gain of chromosome 5 in the SKNO-1 karyotype. *HPRT1* (chrX) showed depth values (23.2× in G, 15.7× in J) aligned with the expectation for a single-copy X chromosome in a male cell line. *SOD1* (chr21), located near the anellovirus integration site, showed slightly reduced depth in the J subline (31.6×) relative to the G subline (40.0×). Intriguingly, the depth of anellovirus coverage (8.3× in J vs 4.7× in G) was less than expected for single-copy coverage in this clonally heterogeneous cell line (27).

### Quantification of anellovirus in SKNO-1 cell line with ddPCR

To quantify the *Alphatorquevirus* sp. isolate within the SKNO-1 cell line, we performed ddPCR with two primer/probe sets. We used primer/probe set 1 (PP1), designed to target the negative strand within the ORF1 coding sequence, and primer/probe set 2 (PP2), designed to target the positive strand in the 5′ 171 bp duplicated end corresponding to non-coding sequence. The virus copy number was quantified in the G and J sublines, with THP-1 monocytes and 293A kidney cells serving as negative controls. Using *RPP30* as the reference gene (assuming two copies/cell based on PacBio data, Fig. S2), the anellovirus copy number was calculated to be 0.42–0.53 copies/cell in the G subline and 0.54–0.56 copies/cell in the J subline (Fig. S3). No anellovirus was detected in THP-1 or 293A cells.

Next, we verified RNA expression of *Alphatorquevirus* sp. in the G and J sublines using RT-ddPCR with the same primer/probe sets and negative controls as in the ddPCR. Transcripts were detected in both the G and J sublines, with no significant DNA contamination observed in the RNA samples. Relative RNA expression, measured as PP1(ORF1):RPP30, was 9.0 in the G subline and 7.5 in the J subline, while PP2(non-coding):RPP30 expression was 3.8 in the G subline and 3.4 in the J subline (Fig. S3). These results suggest that viral transcripts are generated from ORF1 and that a lower level of transcription also occurs from the non-coding viral sequence within the rDNA locus. As expected, no anellovirus transcripts were detected in THP-1 or 293A cells.

### ChIP-Seq and RNA-Seq data analysis of integrated Anellovirus

**Fig 3:**
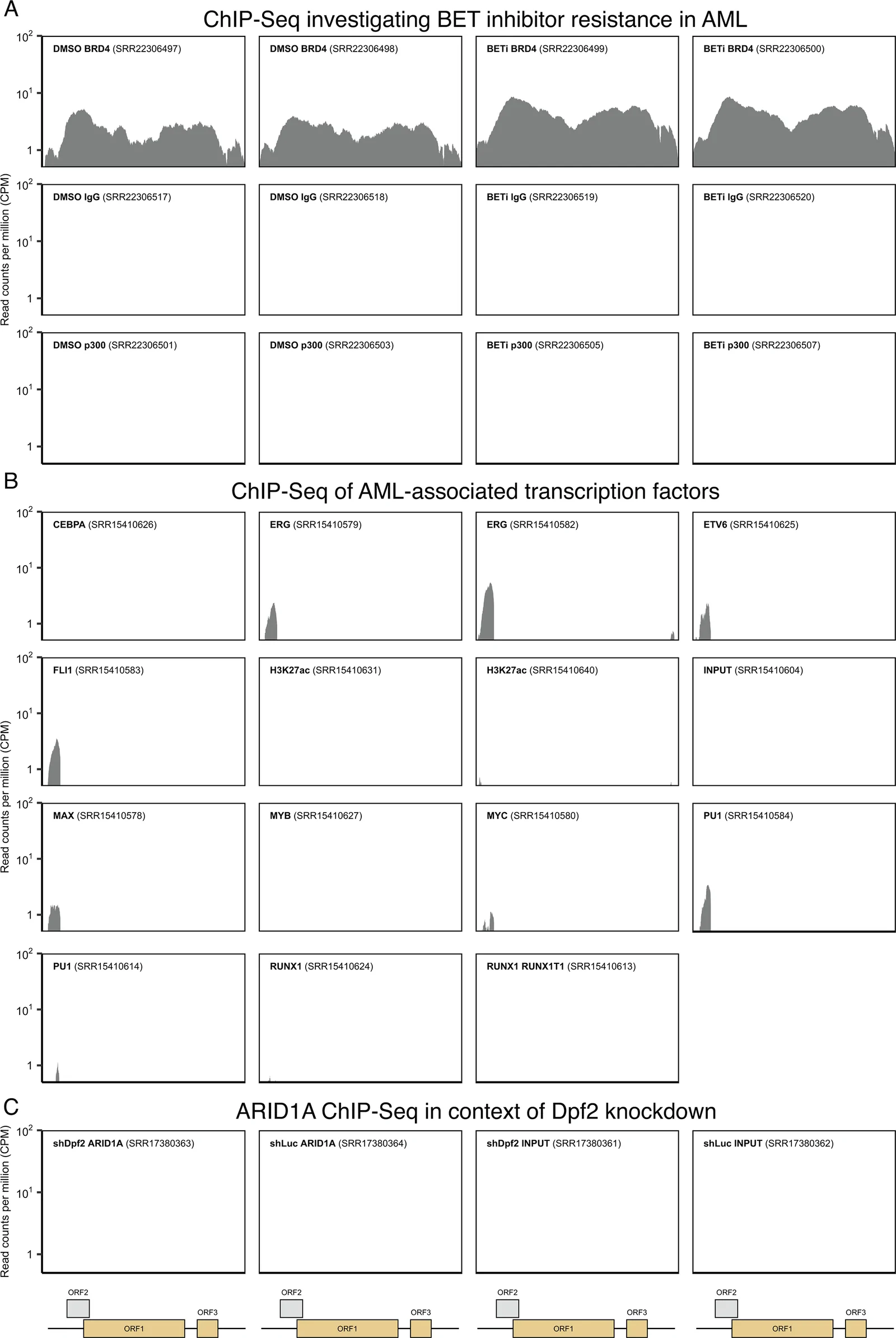
ChIP-Seq SRA data mapping to anellovirus SKNO-1 assembly. Reads from ChIP-Seq SRA data sets were trimmed with fastp and aligned to a *de novo* assembly of the anellovirus genome constructed from RNA-Seq data of the SKNO-1 cell line (see Materials and Methods). Alignment depth is plotted across the 3,425 bp reference genome. Plots are organized into subpanels (**A–C**) labeled with the associated SRA and ChIP-Seq experimental conditions (e.g., protein targeted, buffer). The predicted open reading frame coordinate intervals of the anellovirus assembly, shared across all panels, are displayed once at the bottom of the figure. The span of the entire assembly is depicted by the black bar, and ORFs are shown as yellow boxes.

Next, we used the assembly of the viral-host integrant sequence from the long-read PacBio sequencing as a reference for analyzing a variety of SKNO-1 RNA-Seq (Fig. S4 and S5) and ChIP-Seq data sets (Fig. 3; Fig. S6). RNA-Seq data showed sequencing coverage across the integrant sequence, including the viral ORFs and intergenic regions. Assemblies also showed reads supporting canonical splicing within ORF2 of SKNO-1 anellovirus mRNAs to create ORF2/2 and ORF2/3 splice variants (Fig. S4).

ChIP-Seq data from studies that examined the response of SKNO-1 cells to BET protein inhibition showed that BRD4, a bromodomain-containing protein that canonically facilitates transcription by recruiting transcriptional machinery to acetylated chromatin, binds broadly across nearly the entire *Alphatorquevirus* genome (Fig. 3) (30). Notably, BRD4 occupancy at the viral integration site persisted and even slightly increased following BET inhibition, indicating partial or full resistance to bromodomain inhibition, while p300 chromatin occupancy was minimal. In addition, treatment with BET and p300 inhibitors and selection of drug-resistant SKNO-1 cells increased overall anellovirus transcription by 2- to 4-fold (Fig. S5). This suggests either non-canonical recruitment of BRD4 to the anellovirus locus or its maintenance within a stable, enhancer-like chromatin domain, consistent with an active and accessible regulatory region.

Focal enrichment was observed for hematopoietic transcription factors MAX and the ETS family (ERG, FLI1, SPI-1/PU.1, and ETV6) within the ~300 bp region upstream of the viral open reading frames (Fig. 3) (31). The two tagging SNVs in the 171 bp repeat region (c150a and g162a) demonstrated that only the 5′ repeat was recovered in the transcription factor ChIP-Seq reads. High-scoring motifs were found in the first 312 bp of the viral integrant for human SPI-1/PU.1, MAX, ETV6, and FLI1 transcription factors using FIMO v5.5.9 analysis from the MEME suite (*P* < 0.0001, Table S6) (35), while the ERG motif had a >0.88 relative profile score using JASPAR2026 (Table S7) (36). Interestingly, no H3K27ac enrichment was observed, supporting the non-canonical BRD4 recruitment described above. These data indicate that the integrated viral DNA contains discrete regulatory elements that result in transcription factor binding within a broadly accessible BRD4-enriched chromatin domain.

### Phylogenetic analysis and characterization of integrated SKNO-1 *Alphatorquevirus*

The SKNO-1 anellovirus genome, integrated into chromosome 21, is 3,425 bp in length with a GC content of 50.6%. BLAST analysis identified *Alphatorquevirus homin29* as the closest known viral species, sharing 73.6% nucleotide identity with the ICTV reference genome Torque teno virus 29 (AB038621.1, length 3,676 bp) (37). Notably, the last 316 nt of the Torque teno virus 29 genome are homologous to the first 316 nucleotides of the SKNO-1 anellovirus integrant, while the first 422 nucleotides of the Torque teno virus 29 genome were essentially absent from the SKNO-1 anellovirus, and no homologous sequences were found in the SKNO-1 anellovirus genome by dot plot analysis.

We predicted ORF1 of the SKNO-1 anellovirus using an alternative ACG start codon based on homology with Torque teno virus 29, yielding 80.8% nucleotide identity and 88.9% amino acid similarity (BLOSUM62). SKNO-1 ORF1 contains an early stop codon at Trp565*, producing a protein truncated by 171 amino acids relative to its homolog. The predicted ORF3 shows 87.5% nucleotide identity and 82.6% amino acid similarity. In contrast, the SKNO-1 anellovirus ORF2 contained a mutated start codon (ATG > ACT) and a stop codon at amino acid position 2 and showed limited similarity in the first 18 nt with the Torque teno virus 29 ORF2, suggesting that SKNO-1 anellovirus ORF2 either is not expressed or may initiate translation from an in-frame alternative CTG start at codon 7 of the Torque teno virus 29 ORF2.

**Fig 4:**
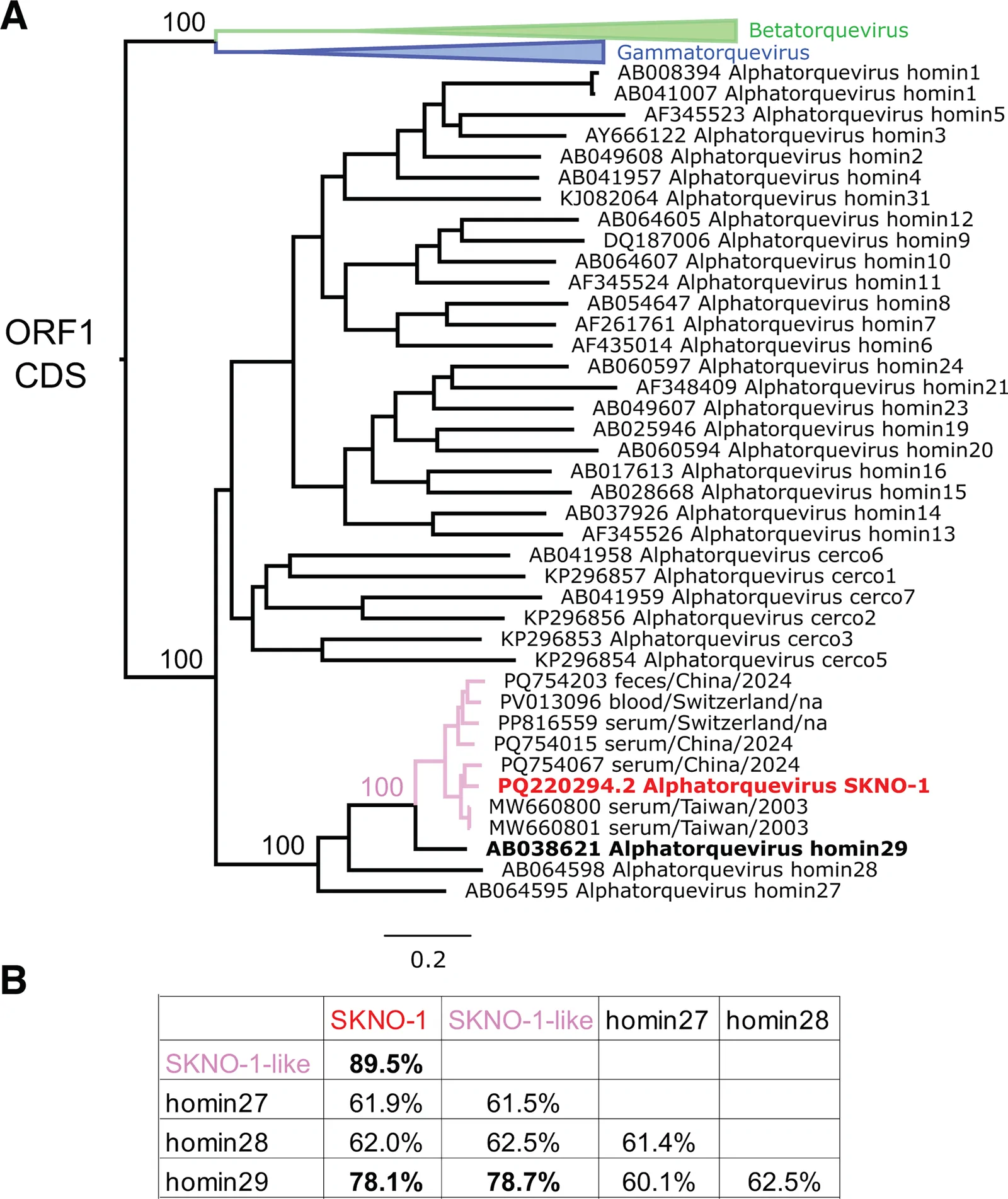
Phylogenetic analysis of anellovirus. (**A**) Maximum-likelihood phylogenetic tree of ORF1 nucleotide sequences from *Alphatorquevirus*, *Betatorquevirus*, and G*ammatorquevirus* reference genomes. Ultrafast bootstrap support values (1,000 replicates) are shown for key nodes. Anellovirus assembled from the SKNO-1 cell line is highlighted in red, and branches corresponding to the clade containing this virus together with sequences retrieved from NCBI BLAST showing >90% genomic identity are shown in pink. The *Betatorquevirus* clade is collapsed and represented as a green triangle, while the *Gammatorquevirus* clade is collapsed and represented as a blue triangle. Branch lengths correspond to the number of substitutions per site. (**B**) P-distance matrix comparing the SKNO-1 anellovirus, the SKNO-1–like anellovirus clade, and representative sequences of *Alphatorquevirus homin27*, *homin28*, and *homin29*.

To confirm the genus and species classification of the SKNO-1 anellovirus, we performed pairwise comparisons and phylogenetic analysis of ORF1 sequences from 92 reference genomes across the *Alpha*-, *Beta*-, and *Gammatorquevirus* genera (Fig. 4). The phylogenetic tree confirmed that the SKNO-1 anellovirus clusters within the *Alphatorquevirus* genus. Pairwise nucleotide comparison showed that SKNO-1 ORF1 exceeds the 69% nucleotide identity threshold relative to *Alphatorquevirus homin29* (AB038621.1), either individually or within its closest evolutionary clade, supporting its classification within the species *Alphatorquevirus homin29* (28).

ORF1 encodes the longest viral protein and functions as the capsid protein (38). In SKNO-1, ORF1 is predicted to produce a 564-amino acid (aa) protein initiating from an ACG start codon (39), considerably shorter than the average *Alphatorquevirus* ORF1 (749 aa) and even shorter than the *Betatorquevirus* ORF1 (664 aa). Due to a lack of experimental validation of the homology-predicted ACG start codon in this *Alphatorquevirus* clade, we also considered an alternative ORF1 prediction of 476 aa initiating at an in-frame ATG codon.

**Fig 5:**
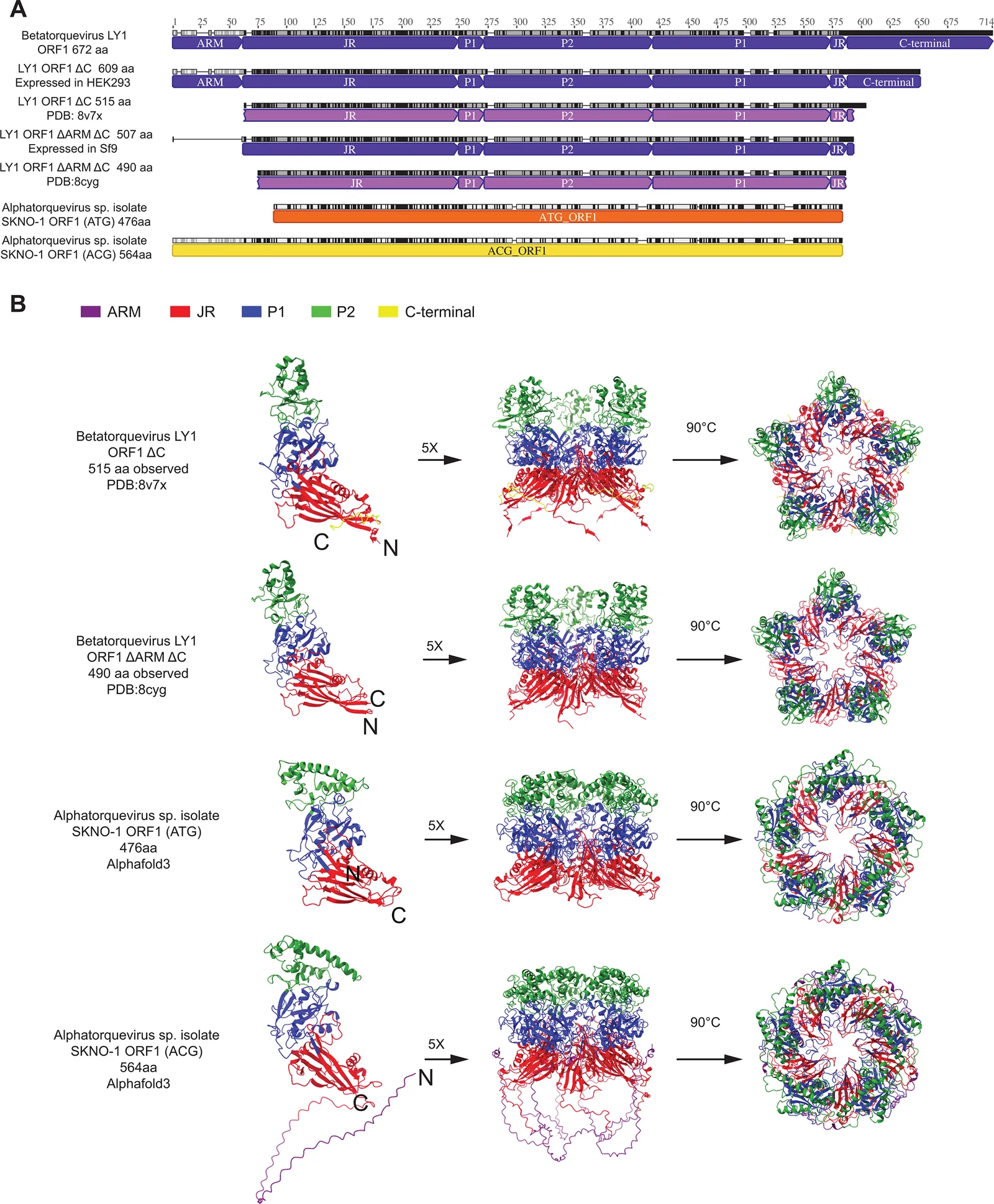
*Alphatorquevirus* sp. isolate SKNO-1 ORF1 (capsid protein) analysis. (**A**) Multiple alignment of amino acid sequence between several *Betatorquevirus* LY1 ORF1 sequences and *Alphatorquevirus* sp. isolate SKNO-1 ORF1 starting with ATG or ACG. The identical sites are marked in black above each motif diagram. The *Betatorquevirus* LY1 ORF1 sequences are annotated with purple bars, and the *Alphatorquevirus* sp. isolate SKNO-1 ORF1 sequences are annotated with orange (ATG) or yellow (ACG) bars. (**B**) The PDB structures of *Betatorquevirus* LY1 ORF1 and the AlphaFold-predicted structure of *Alphatorquevirus* sp. isolate SKNO-1 ORF1 starting with ATG or ACG are shown. The motifs ARM, P2, P1, JR, and C-terminal are marked on the structure. Pentamer structures are shown on the right.

To investigate the structural features of SKNO-1 ORF1, we performed homology modeling using the only published anellovirus ORF1 structure (*Betatorquevirus* LY1 ORF1, PDB: 8V7X) (38). Alignment indicated that SKNO-1 ORF1 retains the jelly roll (JR), P1, and P2 domains but lacks the C-terminal region (Fig. 5A). Since the LY1 ORF1 structure was determined without the ARM or C-terminus, the truncated C-terminus in SKNO-1 ORF1 is not expected to substantially alter the predicted protomeric structure or the putative pentamer, as confirmed by AlphaFold3 modeling (Fig. 5B). Electrostatic surface analysis further indicates a similar charge distribution between LY1 ORF1 and SKNO-1 ORF1 (Fig. S7), suggesting that biophysical properties are largely conserved despite truncation.

## DISCUSSION

Here, using a combination of large-scale *in silico* screening, long-read sequencing, and molecular validation, we identified a stable integration of an *Alphatorquevirus homin29* genome within the rDNA locus of the AML cell line SKNO-1 (27). The integration replaced a 4.5 kb segment of *RNA45SN2*/*RNA28SN2* on chromosome 21 with a 3.6 kb insert containing a complete viral genome, a short unassigned junction, the MLT1D ERVL-MaLR retrotransposon fragment, and an unclassified sequence. The recurrence of this integration in independently sourced SKNO-1 sublines indicated that this is a genuine event rather than contamination. Quantification by ddPCR measured ~0.5 viral genomes per cell, consistent with a subclonal representation, while RT-ddPCR confirmed transcription of ORF1 as well as the non-coding viral integration-associated flanking repeats, revealing that the complete integrated element and the viral ORF1 were transcriptionally competent. We detected spliced ORF2/2 and ORF2/3 transcripts despite the early stop codon observed in the predicted ORF2 sequence (10). Structural modeling suggested that the truncated ORF1 retains key capsid domains and physicochemical properties, hinting at preserved structural potential despite the probable loss of full virion production (38).

Our data raise a number of intriguing hypotheses around anellovirus biology in humans. The existence of a fossilized anellovirus integrant in a *t* (8, 21) AML cell line—thought to be derived from an early myeloid progenitor cell (40, 41)—suggests that the anellovirus may have been present as an episomal genome in the same early myeloid progenitor at the time of leukemia initiation. Integrated anellovirus DNA has recently been found to be a driver of acute promyelocytic leukemia (42–44), whose leukemia-initiating promyelocyte is one step developmentally downstream of the early myeloid progenitor. It has long been recognized that anellovirus levels are highest in peripheral blood and bone marrow, and it can be detected in both mature lymphoid and myeloid cells (3, 45–48). A simple model for the high prevalence of anelloviruses in blood would be residency within hematopoietic stem cells, and our data suggest a cell of origin that is developmentally one step closer to the long-term hematopoietic stem cell progenitor. Alternatively, the subclonal presence of the anellovirus in the SKNO-1 cell line as measured by ddPCR and copy number in sequencing data argues that the anellovirus integrated prior to leukemic initiation. Anellovirus viral loads may be elevated in blood cancers prior to transplant (46). Nonetheless, the presence of 0.5 viral copies per cell would suggest a relatively early integration event along the course of leukemic expansion. Further work, including FISH analysis and/or single-cell RNA sequencing, is required to confirm the subclonal representation of anellovirus.

To date, relatively little is known about the mechanisms that govern anellovirus transcription. Suzuki et al. (49) and Kamada et al. (50) both mapped promoter and enhancer regions within the upstream untranslated region of alphatorqueviruses, and Suzuki et al. (49) showed that upstream stimulating factor (USF) family proteins bound the TTV promoter and that removal of the USF binding site was associated with reduced promoter activity (49, 50). Since 2004, thousands of new human anellovirus sequences have been recovered, and it is unclear whether these mechanisms are conserved among all anelloviruses. For instance, while the TATA box sequence (TATAA) is present in the same location in *Alphatorquevirus homin27*, *28*, and *29* type species (and mutated to TATTA in SKNO-1 anellovirus), the USF binding site is wholly absent in *Alphatorquevirus homin27*, *28*, and *29* and is also absent in the SKNO-1 anellovirus. Notably, due to the 171 bp repeat, the SKNO-1 anellovirus is missing many conserved sequences in its non-coding region, including the GC-rich hairpins downstream of the origin of replication, as well as the TATA box and a hyperconserved domain that acts as a transcriptional start site (10). In short, this complicates interpretation of the finding that hematopoietic cell transcription factors such as ETS family members specifically bind the SKNO-1 anellovirus untranslated region sequence upstream of where viral gene transcription initiates. In addition, the diversity of the anellovirus family and the limited number of well-characterized reverse genetics systems, especially with regard to the ACG-initiating ORF1 human anellovirus species, also complicate sequence homology-based analysis and the functional validation of its predictions. Certainly, additional functional studies are necessary to ascertain whether the transcription factors detected to bind the integrated anellovirus sequence in this study genuinely mediate viral transcription.

The mechanism by which the SKNO-1 anellovirus integrated into the human genome is a matter of speculation. The presence of the 171 bp viral integration-associated flanking repeats in the integrant suggests that the integration happened as part of a viral DNA concatemer, a potential replication intermediate. How circular anelloviruses replicate has long been the subject of speculation, as they encode no Rep protein and there is no prior evidence of long viral concatemers as replication intermediates, as seen in other viruses with circular DNA genomes. Recently, the viral ORF2/3 protein was shown to initiate viral replication and to recruit the eukaryotic polymerase alpha along with the BTR complex, indicating that anelloviruses may use a recombination-dependent mechanism of replication (10). Viral genome concatemers have been used in reverse genetics systems for anelloviruses to initiate viral replication (51), although concatemers have not necessarily been seen in natively infected cells. Alternatively, the viral integration-associated flanking repeats may have been generated not as a concatemer replication intermediate but as part of the recombination-dependent replication process, which subsequently enabled integration. The integration may have arisen via GGGG microhomology-mediated end joining with template switching, a mechanism analogous to those described for hepatitis B virus, human herpesvirus 6A, and human papillomaviruses (52–55). While alternative scenarios—such as prior MLT1D ERVL-MaLR insertion or non-homologous end joining followed by recombination—cannot be excluded, they are less parsimonious. Of note, there are more than 20,000 copies of the MLT1D repeat in the human genome, the repeat fragment contained in the integrant comprises less than half of a full MLT1D repeat, and the MLT1D repeat is present on the opposite side of the anellovirus integrant from where transcription initiates, so this aspect of the integration may be due to chance (56). It is also unclear which RNA polymerase is responsible for the anellovirus transcription. The viral sequence integrated into a transcriptionally permissive locus within an RNA Pol I-transcribed rDNA unit. This region exhibits broad BRD4 occupancy and focal binding by hematopoietic transcription factors MAX and ETS (ERG, FLI1, SPI-1/PU.1, ETV6), which are typically associated with RNA Pol II transcription. Persistent BRD4 engagement despite BET inhibition suggests bromodomain-independent tethering, potentially with coactivators retained through protein–protein interactions rather than high histone acetylation density, consistent with absent H3K27ac ChIP-Seq enrichment, as has previously been described for BRD4 (57–59). Future work involving ChIP-Seq for RNA Pol occupancy or nascent RNA labeling is required to understand whether these features create an “RNA Pol II island” within the nucleolar organizer region, where local chromatin remodeling and sequence-specific elements facilitate transcription of the integrated viral genome, in line with RT-ddPCR detection of both ORF1 and the non-coding viral region.

The presence of the anellovirus integrant in different SKNO-1 sublines and high similarity (>90%) to other *Alphatorquevirus* genomes detected in human clinical samples (collected in 2003 and 2024) suggest that it occurred *in vivo* during leukemogenesis and represents a contemporary viral variant. The subclonal nature of the integrant and its long-term persistence in culture suggest cellular tolerance rather than a selective advantage. Although anelloviruses are typically non-integrative, sporadic integration events have been described, and recent reports suggest that host DNA repair pathways can occasionally capture episomal anellovirus DNA (44). These observations support a model in which the SKNO-1 integrant represents a stochastic, repair-mediated event rather than a biologically selected insertion. Thus, this case likely represents a rare example of somatic viral capture with minimal phenotypic impact, mechanistically analogous, but not functionally comparable, to the repair-driven integrations seen with other non-integrative DNA viruses (52, 54).

This study has a number of limitations. While SKNO-1 provides a striking experimental case of viral integration into human DNA, it does not recapitulate natural anellovirus infection biology in humans, where the virus exists as an episome and persistence would not require integration. While transcriptional activity of the anellovirus locus is evident, production of protein or infectious virions remains unconfirmed. To date, no one has seen bona fide ORF1 protein that includes the conserved N-terminal arginine-rich motif from *Alphatorquevirus* species annotated with an alternative ACG start codon. Future work could use ribosome profiling of SKNO-1 cells and/or these *Alphatorquevirus* species to determine ribosomal occupancy of anellovirus transcripts and whether ORF1 protein is produced. As stated above, we do not know if the ETS family transcription factors are required for SKNO-1 anellovirus transcription, which would require specific depletion and examination of SKNO-1 anellovirus expression. Taking into consideration the cellular transcriptional context is important as well. For instance, we note that RUNX1 was present at similar levels to the ETS family transcription factors in the peri-anellovirus locus, though it was not enriched in the anellovirus locus.

In summary, this study highlights the power of public sequencing data as well as the plasticity of anellovirus genomes and their capacity to integrate into transcriptionally active host loci. The SKNO-1 integration demonstrates how anelloviruses can be tolerated by host cells over long-term culture, offering a unique model to explore, in this understudied virus, the interplay between viral transcription, chromatin environment, and host regulatory factors. This system provides a foundation for future studies on anellovirus transcriptional regulation, epigenetic interactions, and potential impacts on hematopoietic biology.

## MATERIALS AND METHODS

### Data mining in NCBI SRA Cloud by BigQuery

NCBI SRA accession numbers containing reads taxonomically classified as *Anellovirus* (taxid: 687329) were identified using the NCBI SRA Taxonomic Analysis Tool on BigQuery (https://www.ncbi.nlm.nih.gov/sra/docs/sra-cloud/). This data set is based on k-mer-based classification, typically using 32 nt k-mers, performed by NCBI. The list of accessions was obtained on 4 July 2025, using the following command: bq --format=csv query --nouse_legacy_sql --max_rows = 10,000,000 'SELECT acc, total_count FROM `nih-sra-datastore.sra_tax_analysis_tool.tax_analysis` WHERE tax_id = 687,329 and total_count > 1'.

Separately, NCBI SRA runs annotated as derived from human cell lines were retrieved on 4 July 2025, using the following query: bq --format=csv query --nouse_legacy_sql --max_rows = 1,000,000 'SELECT acc, assay_type, jattr, releasedate FROM `nih-sra-datastore.sra.metadata` WHERE organism = "Homo sapiens" and contains_substr(attributes, "cell line")'.

We then identified the subset of human cell line runs that also contained *Anellovirus* k-mer evidence by matching accession numbers across both data sets. These overlapping runs represent cases where anellovirus was detected in human cell line sequencing data, despite not being explicitly reported in the sample metadata. The matching accessions were retrieved with the following command: bq --format csv query --nouse_legacy_sql --max_rows = 10,000,000 'SELECT t1.acc as `Accession Number SRA`, total_count as `Anellovirus K-mer Count`, assay_type as `Assay Type`, TO_JSON_STRING(jattr) as attributes FROM (SELECT * FROM `nih-sra-datastore.sra_tax_analysis_tool.tax_analysis` WHERE tax_id = 687,329 and total_count > 1) AS t1 INNER JOIN (SELECT * FROM `nih-sra-datastore.sra.metadata` WHERE organism = "Homo sapiens" AND contains_substr(attributes, "cell line")) as t2 ON t1.acc = t2.acc. Accession lists for each data set are provided in Tables S1 to S3.

### SKNO-1 RNA-Seq data analysis

From the list of SKNO-1 cell line sequencing containing anellovirus reads, we downloaded three RNA-seq FASTQs with the highest anellovirus k-mer counts, originating from different laboratories (NCBI SRA accession numbers SRR22306486, SRR24085425, and SRR6008465) (Table 1). In addition, RNA-seq data set ERR3003594 was analyzed, as it corresponds to the data set characterizing the SKNO-1 cell line strain obtained from DSMZ repository that was further utilized in this study. Metagenomic analysis was performed by CZID cloud-based metagenomic platform, accessed on 5 July 2025 (34). From each FASTQ file, reads classified as family *Anelloviridae* in CZID were downloaded and *de novo* assembled using SPAdes v4.2.0 with the “isolate” mode and PHRED score offset 33. The longest contig of each *de novo* assembly was chosen for NCBI BLASTn using the non-redundant nucleotide (nt/nr) database. We further evaluated the *de novo* assemblies by mapping the FASTQ reads against the longest obtained contig using minimap2 (version 2.29-r1283) with parameters adjusted for short-read RNA-seq, considering splicing and discarding secondary alignments.

To search for human-anellovirus chimeric reads in SRAs obtained from other cell lines, we first used Kraken2 (v2.17.1) to isolate anellovirus-classified reads (60). To construct the Kraken2 database used for classification, we downloaded all NCBI nucleotide entries under the *Anelloviridae* taxon (ID: 687329) with genome length between 2,000 and 8,000 bp (*n* = 24,398 sequences) and filtered out entries with large homopolymer regions and/or interior sequences homologous to the human genome consistent with misassembly, resulting in 22,255 sequences. Filtering was performed with bbduk.sh from the BBTools suite (v39.26) using the parameters maxns = 0, entropy = 0.955, entropyk = 5, and entropywindow = 100 (61). Similarity to human sequence was manually assessed using BLASTN against *Homo sapiens*. Reads were then classified to a database containing these sequences via Kraken2 with parameters --minimum-hit-groups 2.

Unclassified reads were discarded, and classified reads were aligned to human genome GCA_000001405.15_GRCh38 (comprising chromosomes 1–23, X, and Y, sourced from the analysis set at https://hgdownload.gi.ucsc.edu/goldenPath/hg38/bigZips/analysisSet/) using minimap2 (v2.29-r1283) with parameters -L --secondary no -ax splice:sr and samtools view (v1.21) with parameters -F 2052 (62). As chimeric reads result in only partial alignments, mapped reads were filtered with a custom Python script to have at least 20% of their read length comprised of soft-clipped, unaligned nucleotides. Reads surviving this filter were then realigned to the same *Anelloviridae* reference database used to construct the kraken2 database using minimap2 with the same parameters. To determine if the lack of anellovirus alignment in the samples was due to a lack of coverage, we also *de novo* assembled anellovirus genomes from kraken2-classified reads using spades (v 4.2.0) with parameters --phred-offset 33. Code is available at https://github.com/greninger-lab/anellovirus_in_skno1_paper/.

### SKNO-1 ChIP-Seq data analysis

From the list of SKNO-1 sequencing containing anellovirus reads, we downloaded 10 ChIP-Seq FASTQ files from NCBI SRA. For control purposes, we also downloaded an additional 29 ChIP-Seq SKNO-1 cell line FASTQ files from the same BioProjects but that did not contain anellovirus reads. As most of SKNO-1 ChIP-Seq runs are single-end (SE), we used only R1 reads of the pair-ended runs to keep consistency. Metagenomic analysis was performed as described in the RNA-Seq analysis section. The read coverage generated by minimap2 was then normalized by the total read number to calculate read counts per million (CPM). The sequencing depth profile was visualized in RStudio v2023.06.1 with ggplot2 and Adobe Illustrator 2022.

### Cell culture and collection

Two stocks of the acute myeloid leukemia SKNO-1 cell lines were procured from the DSMZ repository in Germany (G line) and the JCRB repository in Japan (J line). Cells were cultured in RPMI 1640 medium (Life Technologies Corporation, Carlsbad, USA) supplemented with 10% fetal calf serum (Life Technologies Corporation, Carlsbad, USA) and 10 ng/mL recombinant human GM-CSF (Life Technologies Corporation, Carlsbad, USA). THP-1 cells (ATCC, Manassas, USA) were cultured in RPMI 1640 medium supplemented with 10% fetal calf serum. 293A cells (ATCC, Manassas, USA) were grown in DMEM (Life Technologies Corporation, Carlsbad, USA) supplemented with 10% FBS. Cells were kept in a humidifier incubator at 37°C with 5% CO_2_.

### DNA and RNA extraction

Nucleic acid was extracted by adding 0.1 mL chloroform to the cells in 0.5 mL TRI reagent. After vortexing and 5 min of incubation at room temperature, samples were centrifuged at 12,000 × *g* at 4°C for 15 min. DNA was isolated from the interphase and the lower phenol-chloroform phase saved from RNA extraction. DNA was precipitated by adding 0.15 mL of 100% ethanol. After washing twice with 0.1 M sodium citrate in 10% ethanol (pH 8.5) and once with 75% ethanol, DNA was resuspended in 8 mM NaOH. The insoluble materials were removed by centrifuging at 12,000 × *g* at 4°C for 10 min. The pH of the DNA solution was finally adjusted to 7.2 with 0.1 M HEPES.

Carefully moving the aqueous phase into 0.25 mL isopropanol, RNA was precipitated at −80°C for an hour. The precipitated RNA was collected by centrifuging at 12,000 × *g* at 4°C for 10 min. After washing twice with 75% ethanol and air-drying, 45 µL DNase/RNase-free water was added to resuspend the RNA. DNA was digested with 5 µL 10× Turbo DNase Buffer and 1 µL Turbo DNase from the TURBO DNA-free Kit (Thermo Fisher Scientific, Vilnius, Lithuania). After incubating at 37°C for 30 min, the enzyme and divalent cations were removed by adding 5 µL DNase Inactivation Reagent to obtain DNA-free RNA.

### ddPCR and RT-ddPCR assay

Two primer/probe sets were designed using Primer3 based on the complete SKNO-1 anellovirus genome. The first set (PP1) targeted the ORF1 negative strand, with forward primer 5′-TGCCAATCAAGTGGTGGGTT-3′, reverse primer 5′-GAGGGTTTCGTCTGGTCTCG-3′, and probe 5′-/56-FAM/CAATAGTTA/ZEN/ACTGTGGACCGTTTGTCCCAC/3IABKFQ/-3′. The second set (PP2) targeted the positive strand of the 171 bp-duplicated integration sequence, with forward primer 5′-TTAGTGTCTTCCGGGTTCGC-3′, reverse primer 5′-GCGTTTCCTCACCACGTGA-3′, and probe 5′-/56-FAM/CAAGATGGC/ZEN/TGCCGTGACGTCACTAATACG/3IABkFQ/-3′. A reference RPP30 primer/probe set was used, with forward primer 5′-AGATTTGGACCTGCGAGCG-3′, reverse primer 5′-GAGCGGCTGTCTCCACAAGT-3′, and probe 5′-VIC/TTCTGACCTGAAGGCTCTGCGCG-3′.

The ddPCR or RT-ddPCR was performed using the ddPCR Supermix for Probes and One-Step RT-ddPCR Advanced Kit for Probes, as recommended by the manufacturer, including 1 μL of nucleic acid in a final volume of 25 μL (Bio-Rad). For the ddPCR reaction mix, we included 1 μL of HindIII (New England Biolabs). Droplet generation was performed using a Bio-Rad automated droplet generator (Droplet Generation Oil for Probes). Amplification was carried out on a C1000 Thermal Cycler (Bio-Rad) with the following conditions: 95°C for 10 min, 40 cycles of 94°C for 30 s, 60°C for 1 min, and a final 10-min hold at 98°C. Droplets were analyzed on a QX200 or QX600 droplet reader (Bio-Rad) immediately following amplification. Data were processed with QX Manager Software (version 2.1), and quantification was calculated as copies/μL of specimen.

### Long-read sequencing of SKNO-1 cell lines

Frozen SKNO-1 cell pellets (5 × 10^6^ cells each) from the DSMZ and JCRB were submitted to the Translational Genomics Research Institute (TGen) for high-molecular-weight DNA extraction using the NEB Monarch HMW DNA Extraction Kit for Cells & Blood. DNA quality was assessed using the Agilent Femto Pulse, followed by shearing using the Megaruptor 3 (Diagenode). Library preparation was performed using the SMRT-Bell Prep kit V3, and sequencing was carried out on the PacBio Revio system using SMRT-Cell technology, targeting an estimated 40× coverage for the human genome from each cell line.

Long reads were initially mapped to the *de novo* assembled SKNO-1 anellovirus isolate using minimap2 version 2.29-r1283 with the preset “-ax map-hifi” for high-fidelity reads from PacBio. Supplementary and unmapped reads were filtered out with samtools version 1.21 (62). Reads were then aligned to the human genome GRCh38 analysis set (GCA_000001405.15, https://hgdownload.soe.ucsc.edu/goldenPath/hg38/bigZips/analysisSet/hg38.analysisSet.chroms.tar.gz). Mapping of long reads to the human genome was performed using minimap2 with the preset -ax map-hifi and the following parameters: -t 10 -L --no-supplementary -O 3,18 -E 2,1, adjusting gap penalties to improve indel resolution. Only primary alignments were retained for downstream analysis. Depth of coverage for mapping assemblies and specific genes was recorded using PanDepth version 2.26 (63).

### Motif search and statistical analysis

Transcription factor binding motifs within the integrated viral genome were identified using FIMO v5.5.9 (2025) within the MEME suite (35, 64) and relative score analysis using the JASPAR database v11 2026 (36). We downloaded the position-weighted matrices for human transcription factors MAX, SPI-1/PU.1, ERG, FLI1, and ETV6 from JASPAR2026 and input them into FIMO v5.5.9 (https://meme-suite.org/meme/tools/fimo), along with the first 312 bp of the anellovirus integrant, using a threshold *P*-value < 10^−4^. JASPAR analysis was conducted using the same position-weighted matrices and 312 bp anellovirus sequence with the scan option (https://jaspar.elixir.no/) and a relative profile score threshold of >80%. Full results for each analysis are presented in Tables S6 and S7.

### Phylogenetic analysis

We used reference anellovirus genomes provided by ICTV (https://ictv.global/vmr) for phylogenetic analysis. The ORF1 coding sequences of all representative *Alphatorquevirus*, *Betatorquevirus*, and *Gammatorquevirus* species reported by ICTV were downloaded from NCBI GenBank (accessed 2 October 2025). In addition, a set of seven anellovirus genomes with over 90% identity by BLASTn to the assembled SKNO-1 anellovirus genome was also included. Genomes were trimmed to the annotated ORF1 coding sequence and aligned with MAFFT v7.526. ORF1 sequences were also translated based on the standard code using AliView v1.28 and aligned with MAFFT-DASH v7.526. Phylogenetic trees of the nucleotide and amino acid alignments of ORF1 were constructed with IQ-TREE v3.0.1 with 1,000 replicates of ultrafast bootstrap. Trees were visualized with Figtree.

### Protein structure prediction

We downloaded the cryo-EM structures of *Betatorquevirus* LY1 anellovirus virus-like particle expressed in HEK293 from PDB (ID: 8V7X, resolution: 2.80 Å, and 8CYG, resolution: 3.98 Å) (38). In addition, we downloaded the nucleotide sequence of *Betatorquevirus* LY1 ORF1 (NCBI GenBank: JX134044.1) and translated using the standard code. The *Alphatorquevirus* sp. isolate SKNO-1 ORF1 amino acid sequence was translated from the longest coding sequence of the assembled genome. We used the amino acid sequences to predict the structure using AlphaFold3 Server (accessed 17 November 2025) (65). The best prediction model (pTM = 0.84) provided by AlphaFold3 was used for comparative visualization in ChimeraX-1.8 (66).

## Data Availability

NCBI SRA accession numbers analyzed in this study are available in Tables S1 to S3. The sequenced *Alphatorquevirus* sp. isolate SKNO-1 genome was submitted to NCBI GenBank with accession number PQ220294.2. PacBio sequencing reads containing the anellovirus integrant were uploaded to NCBI SRA with accession numbers SRR36563182 and SRR36563183 (BioProject: PRJNA1392521).

## SUPPLEMENTAL MATERIAL

Table S1 spectrum.00904-26-s0001.xlsx SRA sequencing runs containing >1 anellovirus k-mer.

Figure S1 spectrum.00904-26-s0002.pdf Chimeric RNA-Seq reads.

Figure S2 spectrum.00904-26-s0003.pdf Housekeeping gene copy number.

Figure S3 spectrum.00904-26-s0004.pdf ddPCR.

Figure S4 spectrum.00904-26-s0005.pdf RNA-Seq on PacBio assembly.

Figure S5 spectrum.00904-26-s0006.pdf SKNO1 BETi p300 RNA-Seq.

Figure S6 spectrum.00904-26-s0007.pdf ChIP-Seq versus hg38, broader window.

Figure S7 spectrum.00904-26-s0008.pdf Electrostatic analysis of protein.

Supplemental tables spectrum.00904-26-s0009.xlsx Tables S2 to S7.

ASM does not own the copyrights to Supplemental Material that may be linked to, or accessed through, an article. The authors have granted ASM a non-exclusive, world-wide license to publish the Supplemental Material files. Please contact the corresponding author directly for reuse.
