# Supplementary material for: Integration of an anellovirus genome in the SKNO-1 acute myeloid leukemia cell line
Source: Microbiol Spectr. 2026 Aug 31;14(10):e00904-26. doi: 10.1128/spectrum.00904-26 (PMC13689230; doi:10.1128/spectrum.00904-26)

Supplementary Figure 1. Alignment of anellovirus-derived reads from the SKNO-1 RNA-seq dataset (SRA accession ERR3003594), visualized in Geneious Prime using the longest de novo assembled contig as the reference sequence. Individual sequencing reads are displayed in separate rows, with the reference sequence underlined in orange. Nucleotides identical to the reference are shown in light gray. Chimeric reads containing soft-clipped regions with sequence similarity to the human genome are highlighted in dark gray, whereas 5' end soft-clipped regions showing similarity/overlap with the end region of the anellovirus genome are highlighted in light blue.

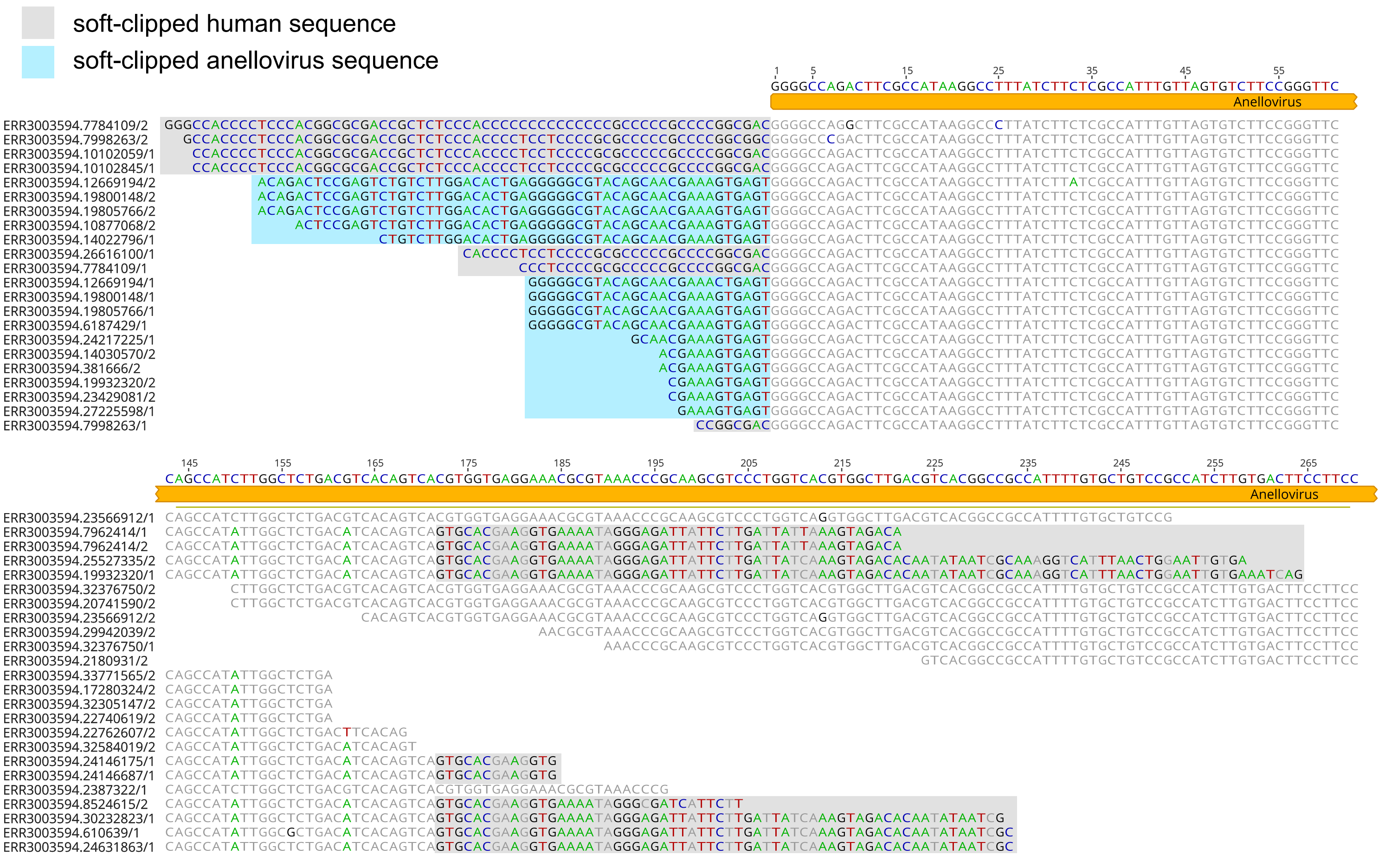

Supplement: Supplementary file 1 [file spectrum.00904-26-s0002.pdf]
