# Supplementary material for: Integration of an anellovirus genome in the SKNO-1 acute myeloid leukemia cell line
Source: Microbiol Spectr. 2026 Aug 31;14(10):e00904-26. doi: 10.1128/spectrum.00904-26 (PMC13689230; doi:10.1128/spectrum.00904-26)

Supplementary Figure 2. Mean sequencing depth of different housekeeping genes, calculated from long-read PacBio sequencing of G and J subline cells containing an anellovirus integrated into chromosome 21. Mean depth was computed as the sum of coverage at each nucleotide position divided by the total length of the gene, using sequencing reads aligned to the hg38 reference genome edited to include the anellovirus integrant in chromosome 21. The SKNO-1 cell line has a complex, clonally heterogeneous human near-diploid karyotype (Matozaki S. et al. Br J Haematol. 1995; DSMZ Cell Line Bank ACC 547).

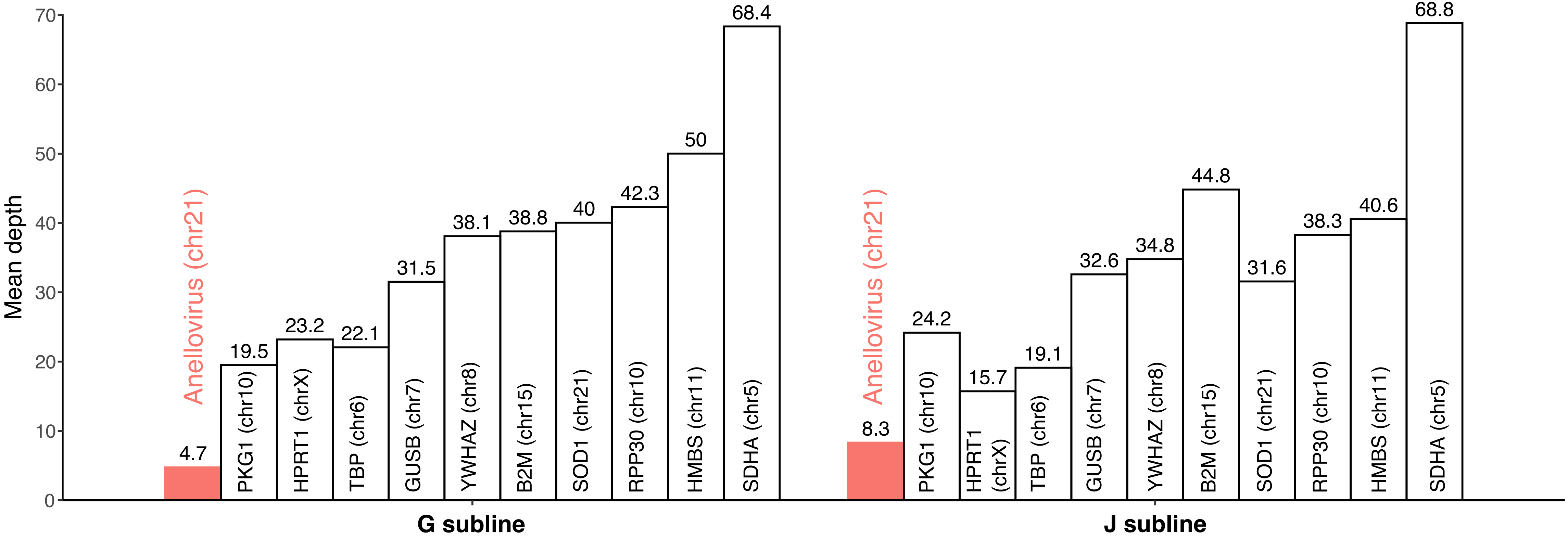

Supplement: Supplementary file 2 [file spectrum.00904-26-s0003.pdf]
