# Supplementary material for: Integration of an anellovirus genome in the SKNO-1 acute myeloid leukemia cell line
Source: Microbiol Spectr. 2026 Aug 31;14(10):e00904-26. doi: 10.1128/spectrum.00904-26 (PMC13689230; doi:10.1128/spectrum.00904-26)

Supplementary Figure 3. Quantification of anellovirus in SKNO-1 cell line using ddPCR and RT-ddPCR.
A) Sequence map of primer/probe sets (PP1 and PP2) used for quantifying anellovirus in SKNO-1 cell line.
B) ddPCR result with PP1 and PP2 on SKNO-1 J and G subline. THP-1 and 293A cell lines are used as negative control. RPP30 gene was used to estimate cell number. Six replicates were performed for each measurement.
C) RT-ddPCR PP1 and PP2 on SKNO-1 J line and G subline. THP-1 and 293A cell lines are used as negative control. Gene expression was normalized to RPP30 transcript levels. Six replicates were performed for each measurement.

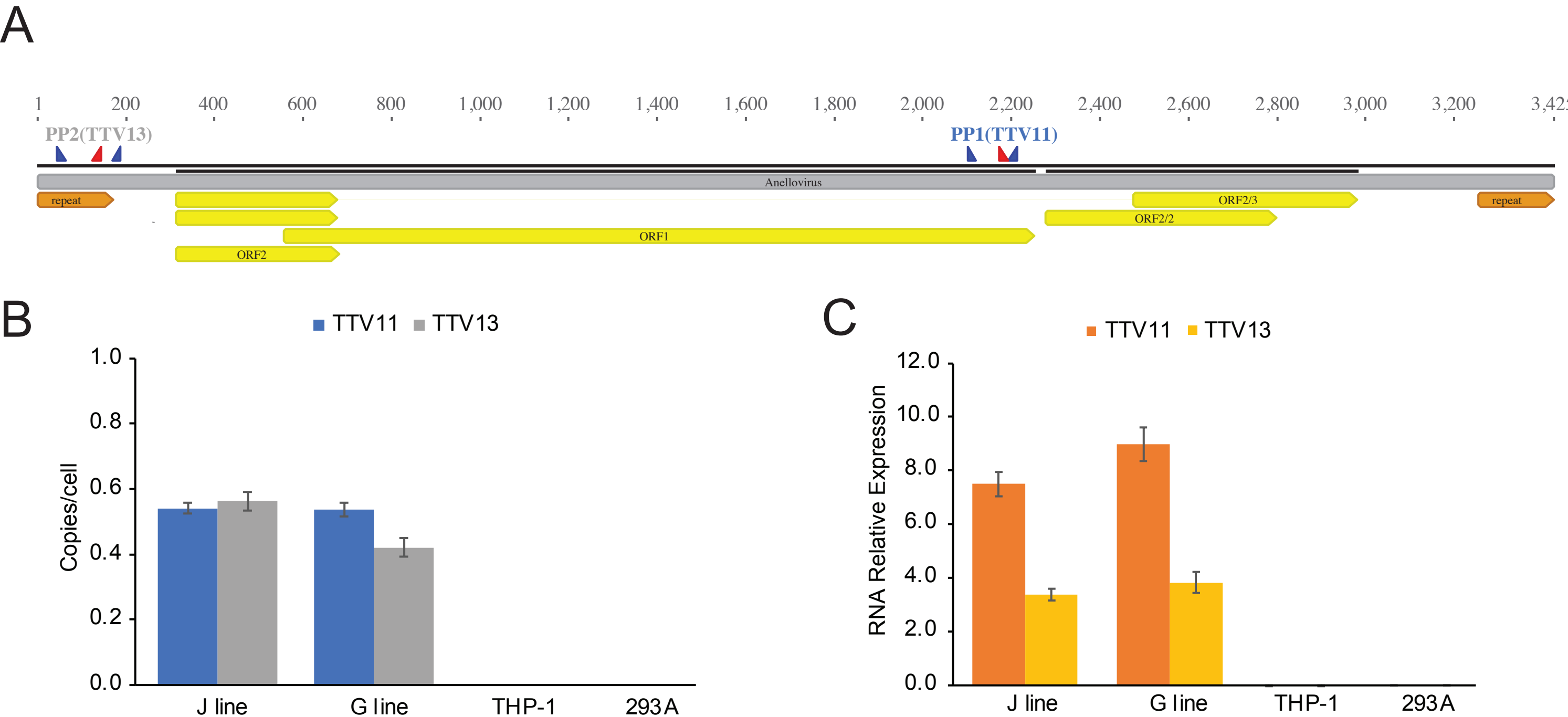

Supplement: Supplementary file 3 [file spectrum.00904-26-s0004.pdf]
