# Supplementary material for: Integration of an anellovirus genome in the SKNO-1 acute myeloid leukemia cell line
Source: Microbiol Spectr. 2026 Aug 31;14(10):e00904-26. doi: 10.1128/spectrum.00904-26 (PMC13689230; doi:10.1128/spectrum.00904-26)

Supplementary Figure 4: RNA-Seq datasets mapped to chr21 with anellovirus integration. Reads from four RNA-Seq SRA datasets were trimmed with fastp and aligned to a chr21 reference modified to contain the integration site of anellovirus in SKNO-1 (see Methods). **A:** Alignment depth across the reference in counts per million (CPM). The coordinate interval containing the anellovirus portion of the integrant is highlighted in red, with the host/chr4 portion of the integrant highlighed in yellow. Plots are labeled with the associated SRA accession. Green brackets represent intronic regions of ORF2/2 and ORF2/3 transcripts. Parentheses: the number of reads with spliced alignments (N in cigar) matching each intronic region. **B:** Visualization of minimap2-generated BAM file for ERR3003594 from panel A showing select reads supporting ORF2/2 and ORF2/3 splicing.

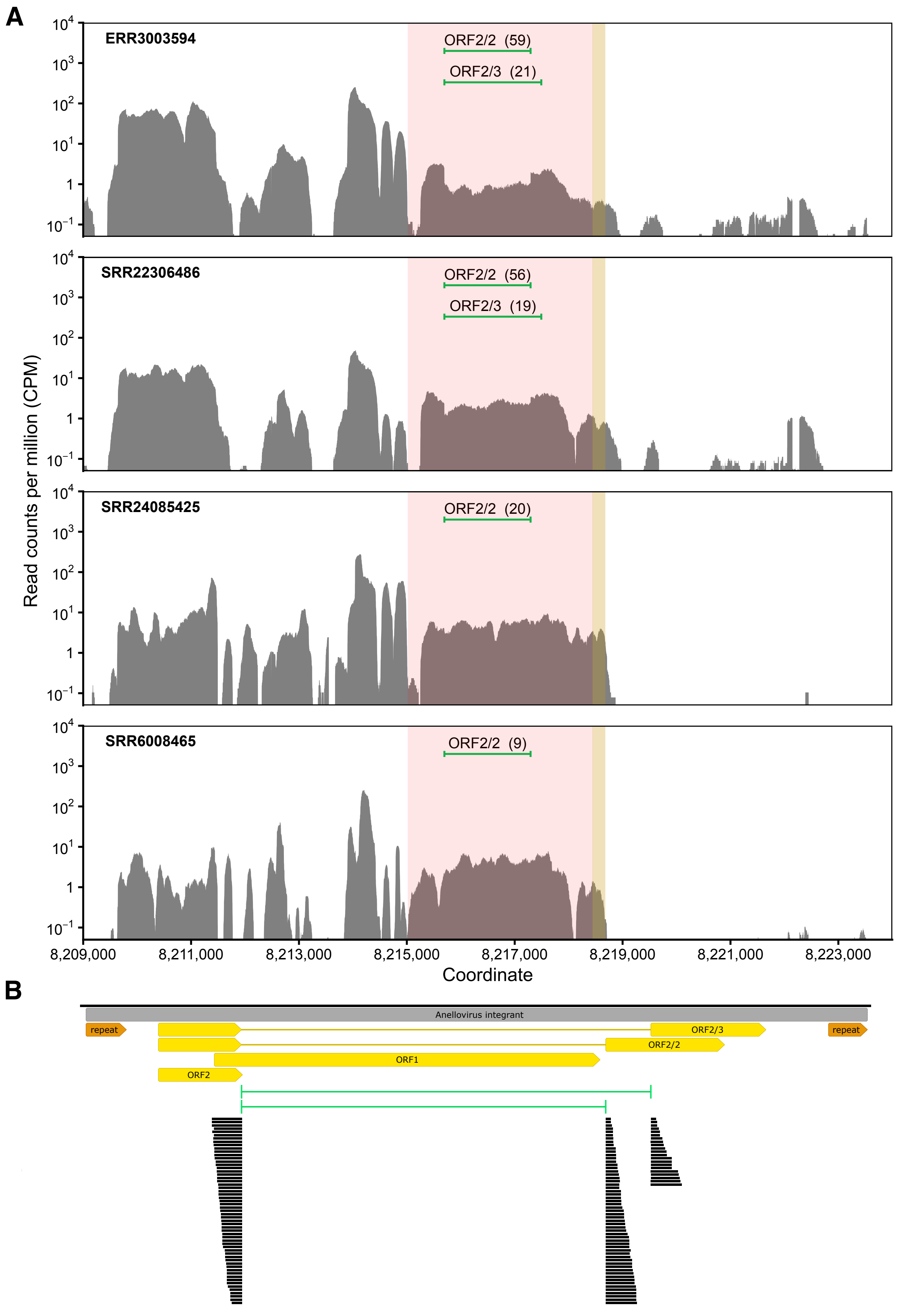

Supplement: Supplementary file 4 [file spectrum.00904-26-s0005.pdf]
