# Supplementary material for: Integration of an anellovirus genome in the SKNO-1 acute myeloid leukemia cell line
Source: Microbiol Spectr. 2026 Aug 31;14(10):e00904-26. doi: 10.1128/spectrum.00904-26 (PMC13689230; doi:10.1128/spectrum.00904-26)

Supplementary Figure 5: SKNO-1 BETi/p300i RNASeq data mapped to hg38 reference containing anellovirus integration in chr21: RNA-Seq datasets from BioProject PRJNA703540 ("Acute resistance to BET inhibitors remodels compensatory transcriptional programmes via p300 co-activation") were trimmed with fastp and aligned to a modified hg38 reference genome containing the anellovirus integration site in chromosome 21 (see Methods). Shown is the alignment depth across the region of chr21 containing the integration. The coordinate interval containing the anellovirus portion of the integrant is highlighted in red, with the host/chr4 portion of the integrant highlighed in yellow. Plots are labeled with the associated SRA and treatment conditions, as well as the mean coverage of the anellovirus region relative to DMSO controls (top row). IC50r and IC90r denote cell cultures with incipient and full-blown BETi resistance, respectively. 3 replicates (each row) are shown per condition.

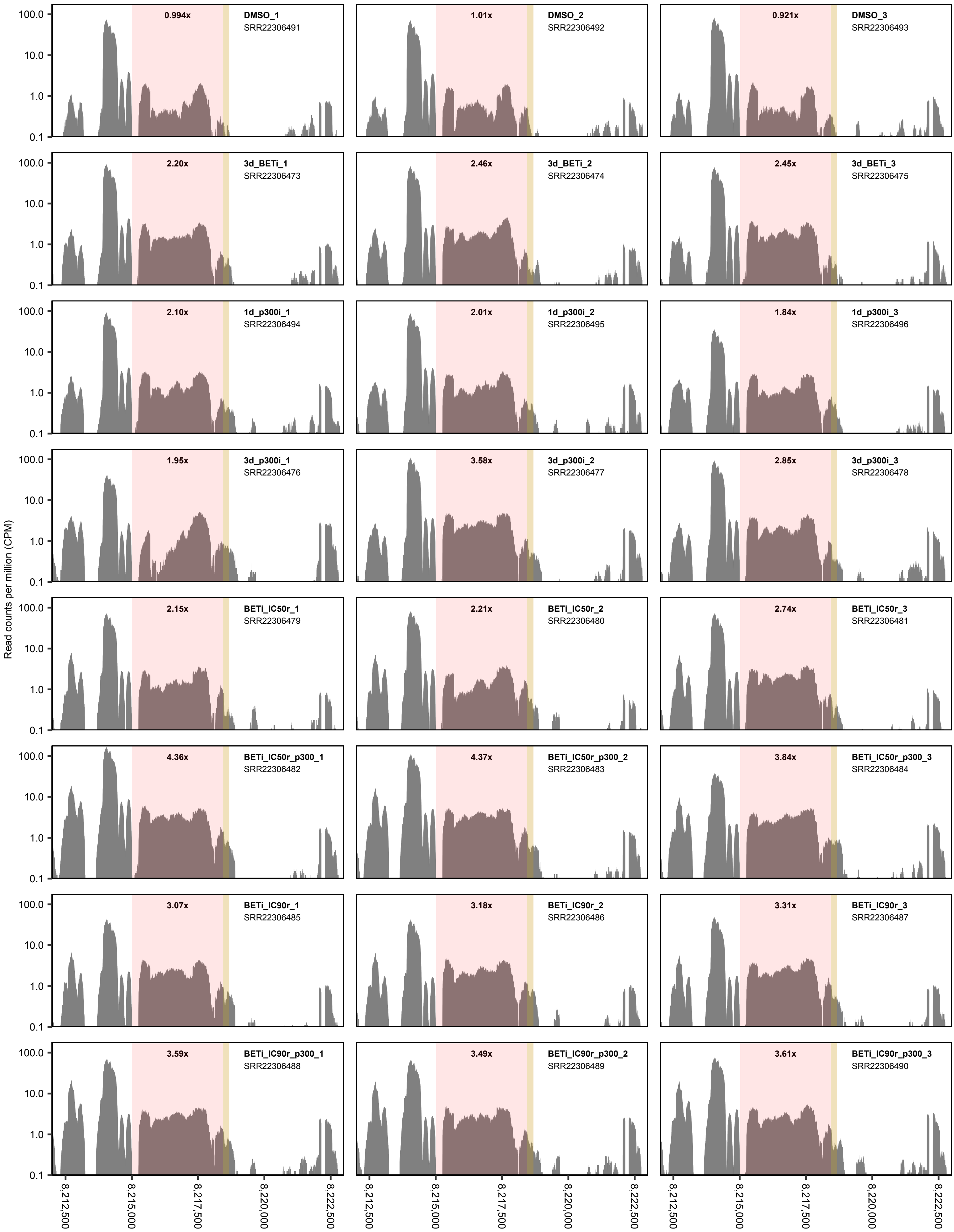

Supplement: Supplementary file 5 [file spectrum.00904-26-s0006.pdf]
