# Supplementary material for: Integration of an anellovirus genome in the SKNO-1 acute myeloid leukemia cell line
Source: Microbiol Spectr. 2026 Aug 31;14(10):e00904-26. doi: 10.1128/spectrum.00904-26 (PMC13689230; doi:10.1128/spectrum.00904-26)

**Supplementary Figure 6: ChIP-Seq SRA data mapped to hg38 reference genome containing anellovirus integrant in chr21:** Reads from ChIP-Seq SRA datasets were trimmed with fastp and aligned to a modified ed hg38 reference genome containing the anellovirus integrant in chromosome 21 (see Methods). Shown is the alignment depth across the region of chr21 containing the integration. The coordinate interval containing the anellovirus portion of the integrant is highlighted in red, with the MalR-like portion of the integrant highlighed in yellow. Plots are labeled with the associated SRA accession and ChIP-Seq experimental conditions.

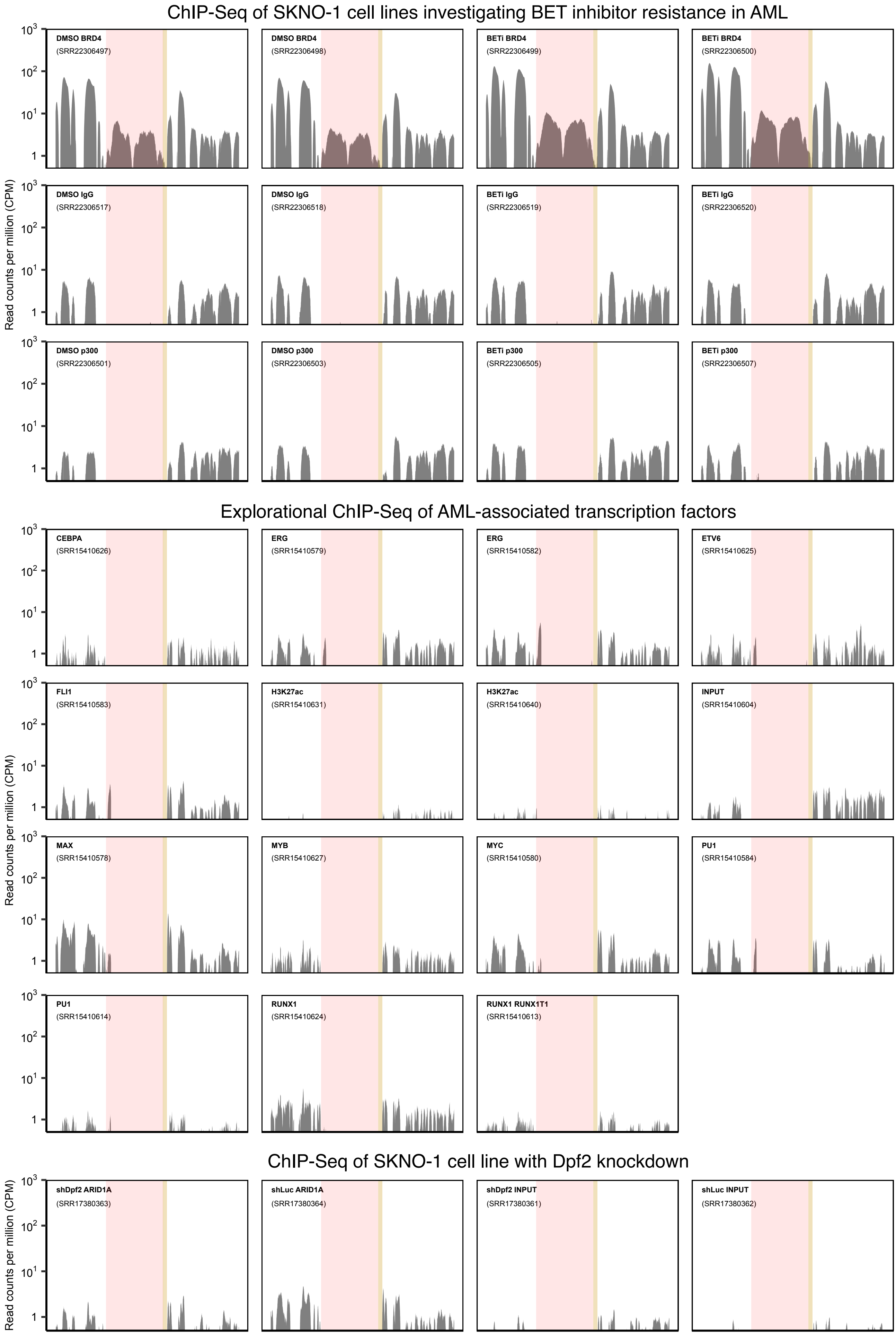

Supplement: Supplementary file 6 [file spectrum.00904-26-s0007.pdf]
