# Supplementary material for: Integration of an anellovirus genome in the SKNO-1 acute myeloid leukemia cell line
Source: Microbiol Spectr. 2026 Aug 31;14(10):e00904-26. doi: 10.1128/spectrum.00904-26 (PMC13689230; doi:10.1128/spectrum.00904-26)

Supplementary Figure 7. Electrostatic analysis of Betatorquevirus LY1 ORF1 ΔARM ΔC and Alphatorquevirus sp. isolate SKNO-1 ORF1. The structures of Betatorquevirus LY1 ORF1 and pentamers are extracted from two PDB models shown on the left. The structures of Alphatorquevirus sp. isolate SKNO-1 ORF1 and pentamers are generated by Alphafold3, given the start codon as ATG or ACG. The motif ARM, P1, P2, JR are marked. The ORF1 monomer is shown in the left column. The lateral view of pentamers are shown in the middle column. The bottom view of pentamers are shown in the right column.

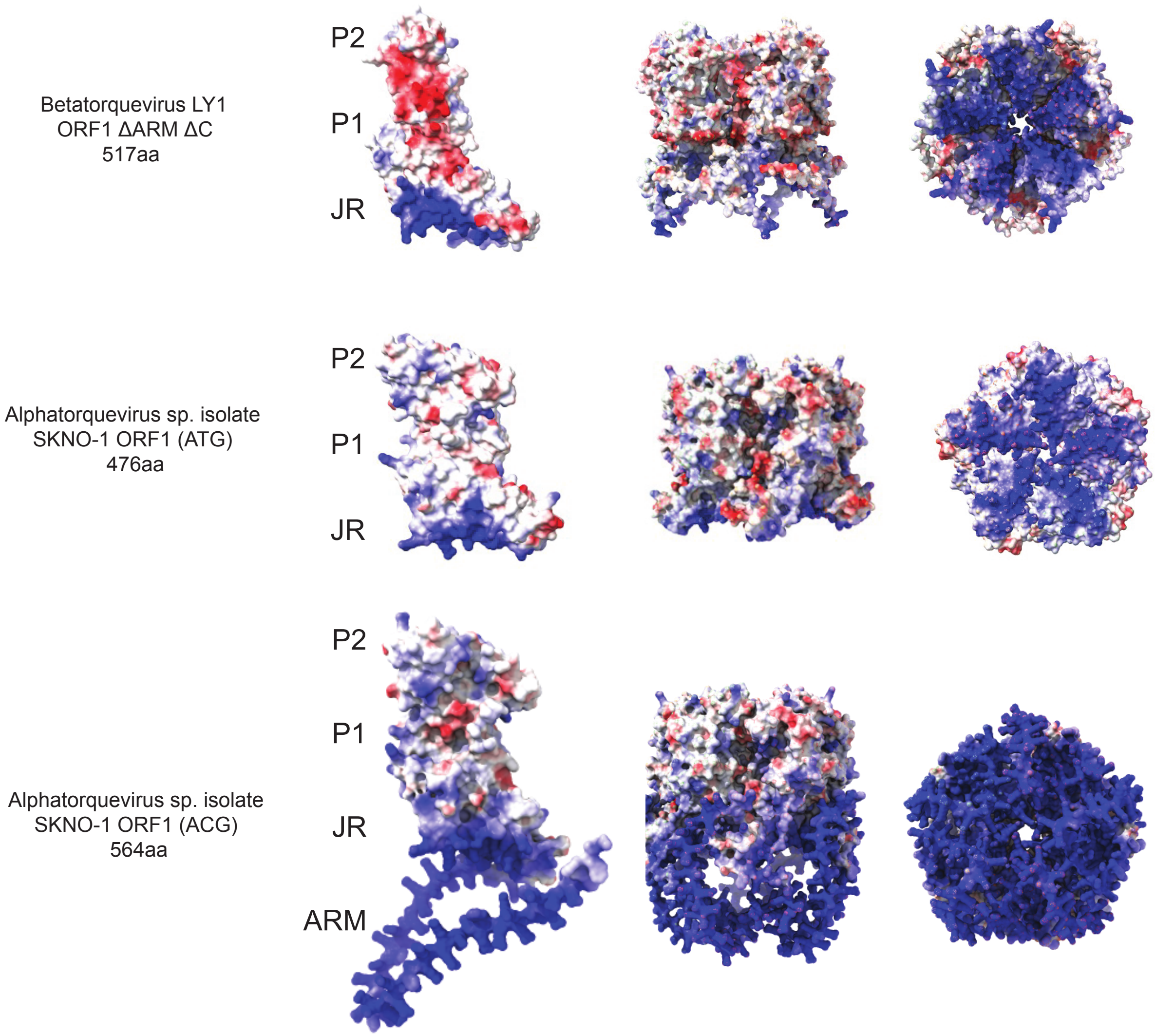

Supplement: Supplementary file 7 [file spectrum.00904-26-s0008.pdf]
